# Supplementary material for: Network-based protein structural classification
Source: R Soc Open Sci. 2020 Jun 3;7(6):191461. doi: 10.1098/rsos.191461 (PMC7353965; doi:10.1098/rsos.191461)
Supplement: Supplementary material for: Network-based protein structural classification [file rsos191461supp1.pdf]

Supplementary material for: Network-based protein structural classification

Khalique Newaz[1,2,3], Mahboobeh Ghalehnovi[1,+], Arash Rahnama[4,+], Panos J. Antsaklis[4], and Tijana Milenković[1,2,3]

[1]Department of Computer Science and Engineering
[2]Interdisciplinary Center for Network Science and Applications
[3]Eck institute for Global Health, University of Notre Dame, Notre Dame, IN 46556, USA
[4]Department of Electrical Engineering
[+]These authors contributed equally to this work

I Supplementary Sections

S1 Protein structure network (PSN) filtering criteria

As described in the main paper, in order to keep only the "meaningful" PSNs for further analysis (except in Section 3(h) in the main paper, where we do not perform this data pruning), we filter the PSNs using an established guideline that is based on network properties of a PSN[1]. Namely, we only keep a PSN if it has 1) a single connected component, 2) a diameter of at least six, and 3) at least 100 nodes (i.e., amino acids). We employ these three criteria for the following reasons (i.e., the following text explains what we mean by "meaningful" PSNs). Below, as an illustration, we use graphlets to explain the validity of these criteria.

We only keep the PSNs with a single connected component for the following reason. The way that we create a PSN for a protein guarantees that the PSN will be connected if each of the amino acids of the corresponding protein is present in its resolved 3-dimensional (3D) structure. This is because we join any two amino acids if the spatial distance between any of their heavy atoms is at least 4Å. Since the distance between two amino acids in the sequence (i.e. a peptide bond) is less than 4Å[2], a protein with all of its amino acids resolved in the 3D structure will be connected. The only way a protein can result in multiple connected components is when there is a large enough number of amino acids that are in its sequence but not in the 3D resolved protein structure. If such proteins are used for PSC, then they will likely bias results, as they are incomplete data. So, in order to avoid such bias in our evaluation due to missing data, we used only those PSNs that have a single connected component.

We only keep the PSNs with at least 100 nodes for the following reason. Graphlets, a state-of-the-art network feature, are local network patterns that capture the global network topology of a PSN. Hence, graphlet-based features for proteins that are too small may not have advantage over simpler network approaches. So, their superiority on more complex topologies with more than 100 nodes might be diminished by their potentially not-as-impressive results on less interesting topologies.

We only keep the PSNs with at least a diameter of six for the following reason. If a protein has all of its amino acids too close in space, then in the corresponding PSN, every node will be connected to almost every other node, which will result in almost a complete network; a complete network is a network in which every node is connected to every other node in the network. In a complete network, whose topology is highly homogenous (with lots of repeating patters of exact same graphlet type, for only few graphlet types), the use of graphlets is not nearly as interesting as in a more heterogeneous network where multiple graphlet types can appear. And restricting the diameter to a minimum of six result in such a network.

S2 Data and protein structure network (PSN) construction

As described in the main paper, following an established guideline[1], we obtain 9,440 and 11,352 PSNs corresponding to CATH and SCOP, respectively. We use these two data sets in our study.

Given the SCOP PSN data, we first test the power of the considered PSC approaches to predict the top hierarchical level classes of the SCOP: *alpha* (α), *beta* (β), *alpha plus beta* (α+β), *alpha/beta* (α/β), *coiled coil*, *membrane*, *multi-domain*, *small*, *low resolution*, *peptide*, and *designed*. For small, low resolution, peptide, or designed, none of the SCOP PSNs belongs to these classes, so we do not consider these classes further. Hence, we take all 11,352 SCOP PSNs and identify them as a single PSN set, where the PSNs have labels corresponding to five top level SCOP classes: α, β,α+β, α/β, and *multi-domain*.

Second, we compare the approaches on their ability to predict the second level classes of the SCOP, i.e., within each of the top-level classes, we classify PSNs belonging to their sub-classes. To ensure enough training data, we focus only on those top-level classes that have at least two sub-classes with at least 30 PSNs each. Five classes satisfy this criteria. For each such class, we take all of the PSNs belonging to that class and form a PSN set, which results in five PSN sets.

Third, we compare the approaches on their ability to predict the third level classes of the SCOP, i.e., within each of the second level classes, we classify PSNs belonging to their sub-classes. To ensure enough training data, we focus only on those second-level classes that have at least two sub-classes with at least 30 PSNs each. Six classes satisfy this criteria. For each such class, we take all of the PSNs belonging to that class and form a PSN set, which results in six PSN sets.

Fourth, we compare the approaches on their ability to predict the fourth level classes of the SCOP, i.e., within each of the third level classes, we classify PSNs belonging to their sub-classes. To ensure enough training data, we focus only on those third level classes that have at least two sub-classes with at least 30 PSNs each. Four classes satisfy this criteria. For each such class, we take all of the PSNs belonging to that class and form a PSN set, which results in four PSN sets.

Thus, in total, we analyze $1+5+6+4=16$ SCOP PSN sets (Supplementary Tables S1-S3).

S3 The Logistic Regression (LR) framework

As outlined in the main paper, given a PSN set, we train an LR classifier corresponding to each of the 34 out of the 35 (all except the weighted network-based feature) pre- or post-PCA protein features (see Section 2(a) in the main paper). Hence, for each of the PSN sets, we get 34 different trained LR classifiers. In each of the trained classifiers, the input is a feature representation of a protein and output is the structural class to which the protein belongs. Below, we outline our LR framework for PSC in detail.

PSC is a multi-class problem. Hence, we use the one-vs-rest scheme to train an LR classifier. That is, given a PSN set with m protein structural classes, we train m different binary LR models that correspond to the m different classes. Each of such binary models can be explained as follows.

Given n augmented feature vectors of the form $x_j = [1, f_1, ..., f_k]$ ($j = 1, 2, ..., n$) of size $K+1$ each, an LR binary model aims to learn optimal values for each of the parameters β_k ($k = 0, 1, ..., K$), in the parameter vector $\beta = [\beta_0, ..., \beta_K]$. In order to determine the optimal values of the β_k parameters, an LR binary model minimizes the cost function $\xi(\beta, x, c)$, where, x is the set of all n data points in a PSN set with the corresponding class labels c. In our study, we use the L_2-Ridge regularized loss function for this purpose (1).

$$\xi(\beta, x, c) = \frac{\lambda}{2}\beta^T\beta + \sum_{j=1}^{n} \log(1 + e^{-c_j \beta^T x_j}). \tag{1}$$

Here, $\frac{\lambda}{2}\beta^T\beta$ is the regularization term that we use to avoid the over-fitting of the model, where λ is the regularization hyper-parameter.

We use liblinear package[3] to minimize the above loss function and learn the corresponding optimal β values for each of the m binary LR models.

So, given a learned LR classifier (i.e., m trained binary LR models), the LR framework estimates the "likelihood" for each of the m classes to be the true label for a test feature vector y using the following equation (2).

$$\sigma(t)_i = \frac{1}{1 + e^{-t}}, \tag{2}$$

Here, $t = <\beta, y>$ is the inner-product of the vectors β (the learned parameter vector for each classifier) and y (the input test feature vector). The output $\sigma(t)_i$ represents the score assigned to the class i ($i = 1, 2, ..., m$) corresponding to the input vector y. We consider the class with highest output score as the predicted class for the given input test vector y.

We use the scikit-learn package in Python[4], in order to implement our LR classifier.

Given a PSN set, we train a logistic regression (LR) classifier corresponding to each of the 34 out of the 35 pre- or post-PCA protein features (see above); we use a different classifier for the remaining (weighted network-based) feature, as discussed in the following subsection. Hence, for each of the PSN sets, we get 34 different trained LR classifiers. In each of the trained classifiers, the input is a feature representation of a protein and output is the structural class to which the protein belongs. Since PSC is a multi-class problem, we use the one-vs-rest scheme to train an LR classifier. Due to space constraints, we provide further details about our LR framework in Supplementary Section S3.

Given a PSN set and a protein feature, we first divide the PSN set into 10 equal-sized subsets, such that each subset contains the same proportion of different protein structural classes (i.e., labels) as present in the initial PSN set. Then, for each subset, we use it as the test data and the union of the remaining nine subsets as the training data. We use the training data in two different ways to train an LR model. First, we train an LR model with the training data as is. We call this way of training an LR model as *proportional*. Second, at least some of our PSN sets have unequal numbers of proteins in different classes, i.e., are imbalanced. Consequently, the training data as used by the proportional approach is also unbalanced. Hence, we exploit a data re-sampling approach called Synthetic Minority Oversampling Technique (SMOTE)[5] to first balance the training data and only then use it to train an LR model. We call this way of training an LR model as *proportional+SMOTE*.

Given a training dataset, we use 10-fold cross validation to choose an optimal value for the hyper-paramter λ. We linearly search an "optimal" value for λ from the set $\{2^{-2}, 2^{-1}, 2^0, 2^1, 2^2\}$. We say that a given value is an "optimal" choice for λ if, on average over 10-folds, the corresponding value results in the best classification performance based on a given evaluation measure. We evaluate the performance of the model using two different popular evaluation measures: accuracy and Matthew's Correlation Coefficient (MCC)[6]. We evaluate the performance of the model on the separate test data using two different evaluation measures: accuracy and Matthew's Correlation Coefficient (MCC)[7]. Accuracy measures the percentage of all proteins from the test data that are classified into their correct protein structural classes. In case of imbalanced dataset, accuracy can give over-optimistic classifier performance as its value is biased towards the class with majority number of samples. That is,

accuracy can fail to capture how a classifier performs on each of the classes present in a dataset. So, we also use MCC, which intuitively captures how well a given classifier performs on each of the classes present in a dataset, and hence is robust to data imbalance. Note that we choose the “optimal” value for λ based on just the training data, which is independent of the test data. We use this “optimal” λ value to train the classifier using all of the training data, and then evaluate the performance of the classifier using a separate test data, using both accuracy and MCC. Because we measure the performance of a trained LR model over 10 different sets of test data, we report an accuracy or MCC performance value averaged over the 10 sets.

S4 The deep learning (DL) framework

In the second part of our study, we design a DL framework, in order to learn features of 3D protein structures using weighted protein structure networks. For each of the 36 PSN data sets, we train a deep neural network, where we use distance matrix-representations of proteins as input.

Our DL framework consists of one input layer, seven hidden layers, and an output layer. Since neural network frameworks can only take as input one dimensional vectors and not the whole distance matrices, we need some way of transforming the distance matrices to one dimensional vectors. A simple way to do this is to flatten a distance matrix, for example, concatenate each row of a distance matrix to one another, and obtain the corresponding one dimensional vector. However, protein distance matrices are of different sizes and hence simple flattening of the matrices would result in vectors of different sizes. This is a problem because a neural network architecture has fixed input layer size.

To overcome this, we use a re-sizing approach, called interpolation, that is commonly used in other research areas, such as, computer vision[8]. Intuitively, given an input data matrix of size $s \times s$, interpolation aims to compress the input matrix into an output matrix of a target size $t \times t$ using a summarizing function. In our study, given a PSN set, we take the size of the smallest protein as the target size with averaging as the summarizing function. We use SKIMAGE package in Python[9] to implement our interpolation procedure.

So, given a PSN set, the size of the input layer for our DL framework is the size of the *flattened* interpolated matrix.

We use seven hidden layers of sizes $1000, 600, 320, 170, 85, 40$, and 12, respectively. Formally, the general model of DL with the parameter set $\theta = [W, B]$ is given by equation (3).

$$Y_\theta(U) = S(WU + B), \tag{3}$$

where, W is the collection of weights $\{W_i\}_{i=1:8}$, B is the collection of biases $\{B_i\}_{i=1:8}$, U is the collection of inputs $\{U_i\}_{i=1:8}$, and Y is the collection of outputs $\{Y_i\}_{i=1:8}$, corresponding to each of the seven hidden layers plus the output layer. The function S represents the activation function called tanh.

Given a hidden layer $i+1$, each of the neurons in the hidden layer produces an output y_{i+1} that is computed as $y_{i+1} = \tanh(\sum_{j=1}^{k} w_j \cdot u_j + b_i)$. Here, k is the total number of neurons in the layer i, u_j is the output of neuron j in the layer i, and w_j is the weight connecting the neuron j in the layer i to the given neuron in the layer $i+1$.

Given a PSN set, we train a DL framework by minimizing the objective function J (4) in the presence of an L_2-Ridge regularization with parameters $\lambda_1 = \lambda_2 = 0.01$.

$$\min J = \arg\min_{w_i, b_i} \{L(W, B|U) + \lambda_1 ||W||_2^2 + \lambda_2 ||B||_2^2\}. \tag{4}$$

Here, the loss function $L(W, B|U)$ is a cross-entropy function and is defined by equation (5).

$$L(W, B|U) = -\sum_{i=1}^{n} \sum_{j=1}^{m} c_{ij} \log \hat{c}_{ij}, \tag{5}$$

Here, n is the number of training data points, c_{ij} and $\hat{c}_{ij}$ are the true and the predicted class labels for a particular input data point, respectively.

We use "Xavier" weight initiation, in order to initialize the training process[10]. Also, in order to minimize the objective function J, we use Adam algorithm, which has been shown to outperform the classical stochastic gradient descent procedure[11].

Given a PSN set, the size of the output layer is equal to the different number of classes present in the PSN set. In the output layer of our DL framework, in order to generate the final classification output as the probability distribution over the classes of a given PSN set, we use Softmax transformation that is defined by equation (6).

$$\sigma(z_j) = \frac{e^{z_j}}{\sum_{j=1}^{m} e^{z_j}}, \quad j = 1, \ldots m, \tag{6}$$

Here, z_j represents the output of neuron j of the output layer without the Softmax function, and $\sigma(z_j)$ represents the probability of class j to be the true class.

Given an input test data point, our DL framework predicts the class with the highest probability as the true class of the input test data point.

We use Python along with the Google's TensorFlow package[12], in order to design our deep learning framework.

S5 Protein class-based performance analysis of our graphlet approaches and GIT

As we outline in Section 3(c) of the main paper, We find that out of all 256 protein structural classes, OrderedGraphlet-3-4 (6Å) outperforms GIT for 84 of the classes (Suppementary Table S4), GIT outperforms OrderedGraphlet-3-4 (6Å) for 132 of the classes, and the two are tied for the remaining 40 classes. That is, OrderedGraphlet-3-4 (6Å) is at least as good as GIT for $124/256 = 48\%$, i.e., almost half, of all classes. It would be interesting to see whether the three sets of classes (those where OrderedGraphlet-3-4 (6Å) is the best, those where GIT is the best, or those where the two are tied) contain different types of protein secondary structural categories. To examine this, for each class set, we compute the enrichment of its classes in each of the level 1 protein structural categories of CATH and SCOP (which we summarize into "α-only", "β-only", "both α and β", and "other" categories); these categories roughly correspond to different types of secondary structural elements.

We first obtain 228 different classes that are present in any of the 35 CATH/SCOP PSN sets that we use in our study (Figure 1 in the paper). Specifically, in order to obtain the 228 classes, we take sum of the number of the classes present in the individual PSN sets. That is, we take all three classes from the CATH-primary PSN set, all seven classes from the SCOP-primary PSN set, all four classes from the CATH-α PSN set, all 10 classes from the CATH-β PSN set,..., and so on, and add them up to obtain 228 different classes. Additionally, we take union of the above 228 classes with 32 classes that are present in the Astral PSN set (Section 2(a) in the main paper), which gives us a total of 256 classes. Note that since the Astral PSN set is essentially a part of the SCOP database, four classes from the Astral PSN set are already present in our previous set of 228 classes and hence when we take the union of 228 and 32 classes then we end up with 256 classes, instead of 228+32=260 classes.

We summarize the 256 classes (using their respective level 1 classes of CATH or SCOP) into "α only", "β only", "both α and β", and "other" classes (Figure 1 in the main paper). That is, we summarize the CATH structural classes 1.10, 1.20, 1 (i.e., α), etc., and the SCOP structural classes a.118, and a (i.e., α), etc. into the class "α only" because they all belong to the CATH/SCOP level 1 class α. Out of 256 classes, we have 48 such "α only" classes. We summarize the CATH structural classes 2.60.40, 2.60, and 2 (i.e., β), etc., and the SCOP structural classes b.1.1, b.1, and b (i.e., β), etc. into the class "β only" because they all belong to the CATH/SCOP level 1 class β. Out of 256 classes, we have 64 such "β only" classes. Similarly, we summarize the CATH structural classes 3.30.390, 3.30, and 3 (i.e., α/β), etc., and the SCOP structural classes c.2.1, c.23, c (i.e., α/β), and d (i.e., α+β), etc. into the class "both α and β" because they all belong to the CATH/SCOP level 1 class α/β or α+β. Out of 256 classes, we have 139 such "both α and β" classes. Finally, we summarize the CATH structural classes that belong to neither of the CATH/SCOP level 1 classes α, β, α/β, or α+β as the class "other". Out of 256 classes, we have five such "other" classes.

Given these 256 classes, we identify classes in which OrderedGraphlet-3-4(6Å) performs the best. Out of 256 classes there are 84 such classes. We summarize these 84 classes in into "α only", "β only", "both α and β", and "other" classes, using the similar procedure as explained above. We obtain 13, 22, 46, and three classes that belong to our classes of "α only", "β only", "both α and β", and "other", respectively. Also, we identify classes in which GIT performs the best. Out of 256 classes there are 132 such classes. We summarize these 132 classes in into "α only", "β only", "both α and β", and "other" classes, using the similar procedure as explained above. We obtain 27, 32, 72, and one classes that belong to our classes of "α only", "β only", "both α and β", and "other", respectively. Similarly, we identify classes in which both OrderedGraphlet-3-4(6Å) and GIT perform the best. Out of 256 classes there are 40 such classes. We summarize these 40 classes in into "α only", "β only", "both α and β", and "other" classes, using the similar procedure as explained above. We obtain 8, 11, 20, and one classes that belong to our classes of "α only", "β only", "both α and β", and "other", respectively.

For each of the three sets of classes (i.e., the set of 84 classes in which OrderedGraphlet-3-4(6Å) performs the best, 132 classes in which GIT performs the best, and the set of 40 classes in which both OrderedGraphlet-3-4(6Å) and GIT perform the best), as is typically done, we use the hypergeometric test[13] to compute whether a set of classes is enriched in any of the classes of "α only", "β only", "both α and β", and "other". Intuitively, given the observed number of occurrences of a class, the hypergeometric test measures the probability of getting the same or higher number of occurrences of the same class in a randomly chosen set of classes of the same size. The latter is randomly selected from a set of background classes; as this set, we use all 256 classes. We say that a class is statistically significantly enriched in a set of classes if its enrichment p-value is ≤ 0.05.

S6 Our features versus SVMfold

SVMfold has high running time because it needs to extract three sets of very comprehensive features from protein sequence information. This complex information retrieval process needs to be performed for each protein in the considered PSN set, which is not feasible when analyzing large PSN sets containing many proteins (such as those at the higher levels of CATH/SCOP hierarchies) or many PSN sets. Hence, we can compare our approaches to the state-of-the-art SVMfold approach only for two representative PSN sets out of all 36 PSN sets.

Specifically, we choose CATH-3.20.20 and CATH-3.40.50 from group 4 of the CATH data as the representative PSN sets, for the following reasons. These two PSN sets correspond to the fourth level of the CATH hierarchy, i.e., as specific structural classes as possible, which are the most relevant for applied biochemistry scientists. Also, of all fourth-level PSN sets, CATH-3.20.20 is one of the PSN sets in which at least one of our top performing graphlet approaches give low accuracy ($\sim$80%), which gives SVMfold the best-case advantage over our approaches, and CATH-3.40.50 is one of the PSN sets in which both of our top performing graphlet features give high accuracy ($\geq$ 95%), which gives our approaches the best-case advantage over SVMfold.

Overall, our best performing graphlet features OrderedGraphlet-3-4 and OrderedGraphlet-3-4(6Å), and our integrated features GIT+OrderedGraphlet-3-4(6Å) and Concatenate are comparable (within $\pm$5%) to SVMfold in terms of accuracy (individual graphlet features on CATH-3.40.50, and GIT+OrderedGraphlet-3-4(6Å) and Concatenate on both CATH-3.20.20 and CATH-2.40.50) at a fraction of SVMfold's running time (Table S13).

II Supplementary Figures

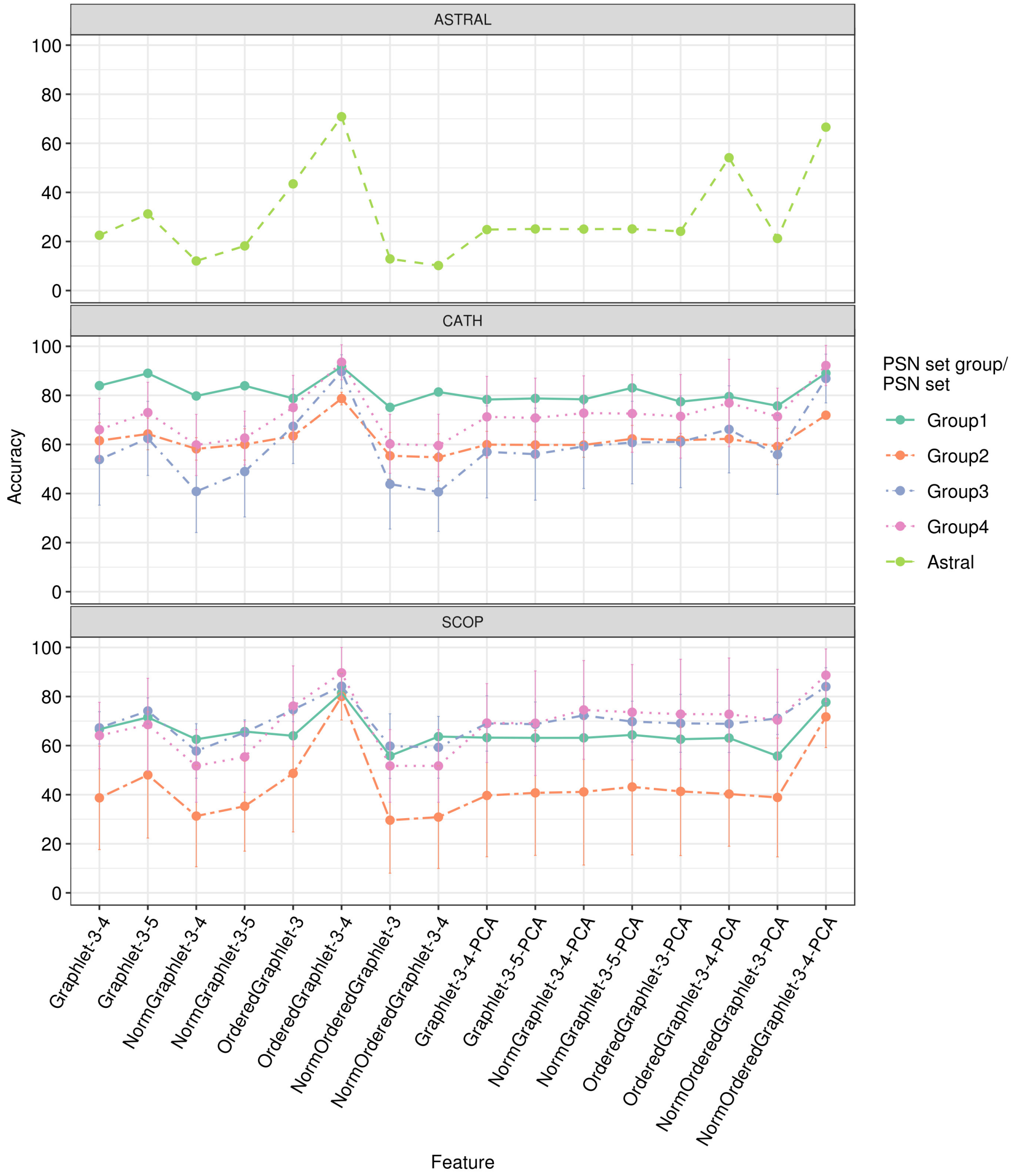

Supplementary Figure S1. Accuracy of the 16 pre- and post-PCA graphlet features under the LR classifier, for ASTRAL PSN set (i.e., group), each of the four hierarchy levels (groups) of CATH, and each of the four hierarchy levels (groups) of SCOP, averaged over all PSN sets belonging to the given group (vertical lines are standard deviations).

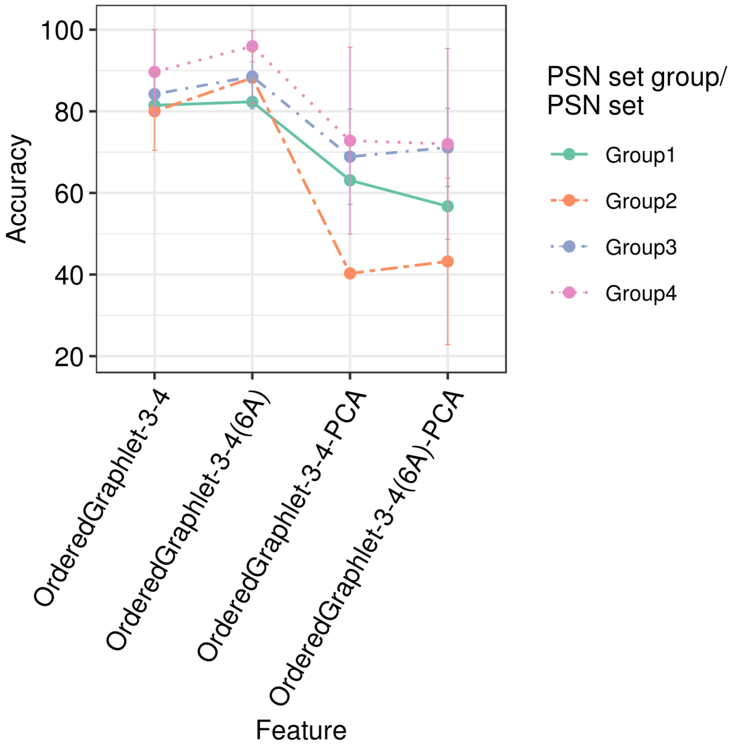

Supplementary Figure S2. Accuracy of the pre- and post-PCA OrderedGraphlet-3-4 (ordered graphlets at the default 4Å distance cutoff) and OrderedGraphlet-3-4(6Å) (ordered graphlets at the 6Å distance cutoff) under the LR classifier, for each of the four hierarchy levels (groups) of the SCOP data, averaged over all PSN sets belonging to the given group (vertical lines are standard deviations).

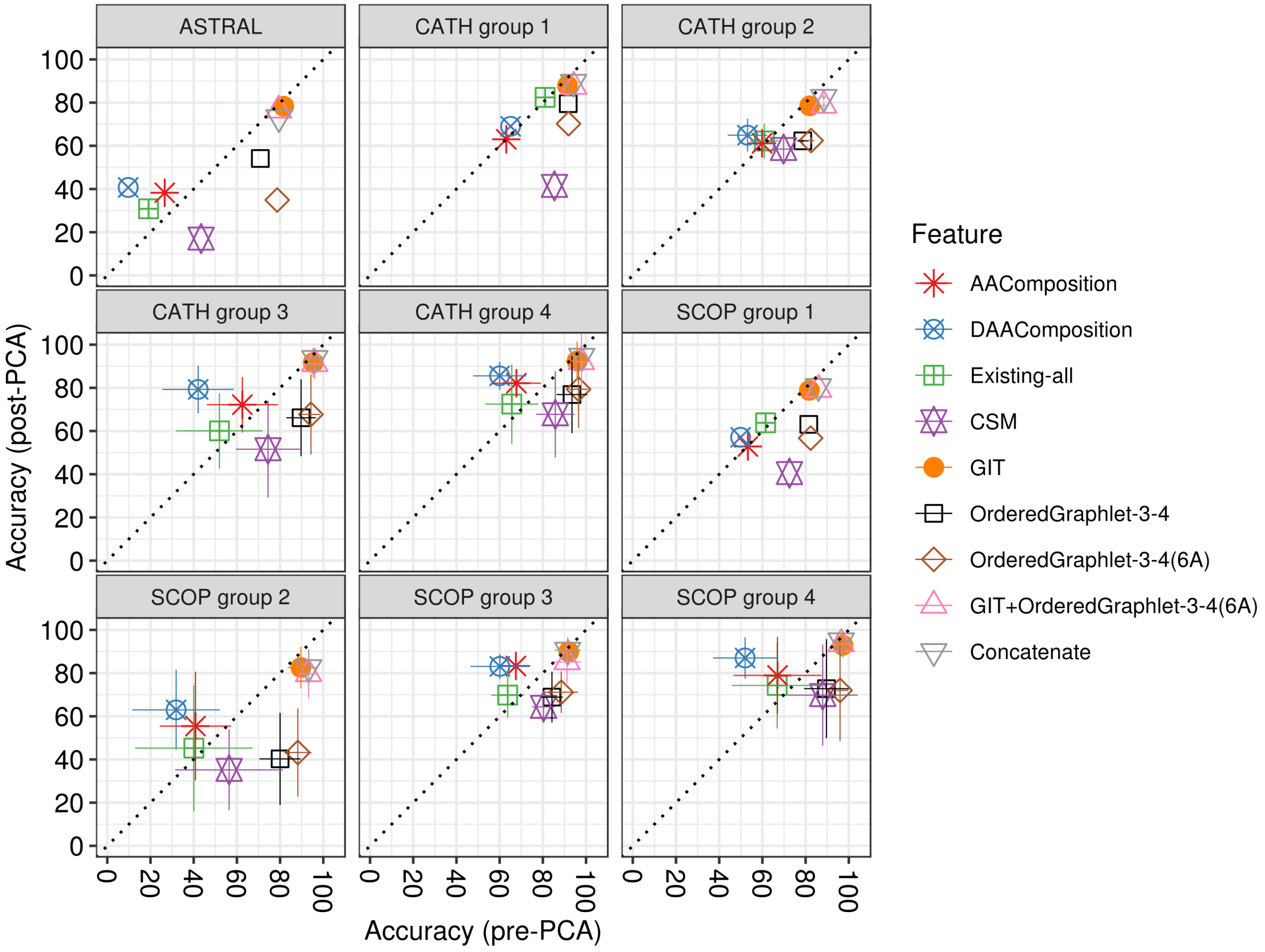

Supplementary Figure S3. Accuracy of pre- and post-PCA versions all non-graphlet features except SVMfold, the two top performing graphlet features from Section 3(a), and all integrated features (i.e., GIT+OrderedGraphlet-3-4(6A) and Concatenate) under the LR classification framework (Table 1 in the paper), for each of the PSN set groups. Results are averaged over all PSN sets in the given group (horizontal and vertical lines are standard deviations).

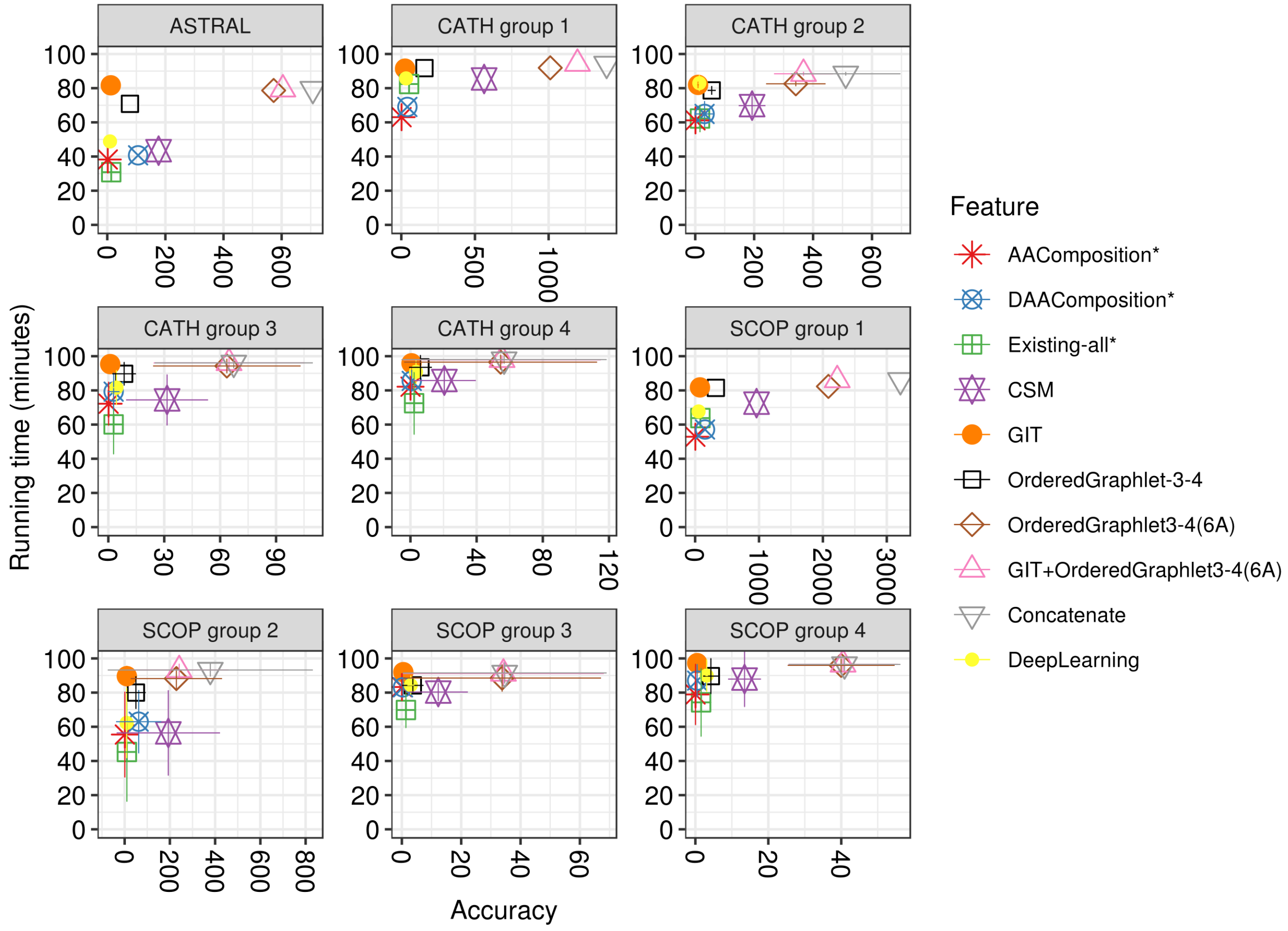

Supplementary Figure S4. Accuracy versus running time of the features from Figure 3 in the main paper, for each of the PSN set groups, under LR framewrok. For each method except DL, the best of its pre- and post-PCA versions is chosen (DL does not have this option). If the latter is selected, "*" is shown next to the given feature's name.

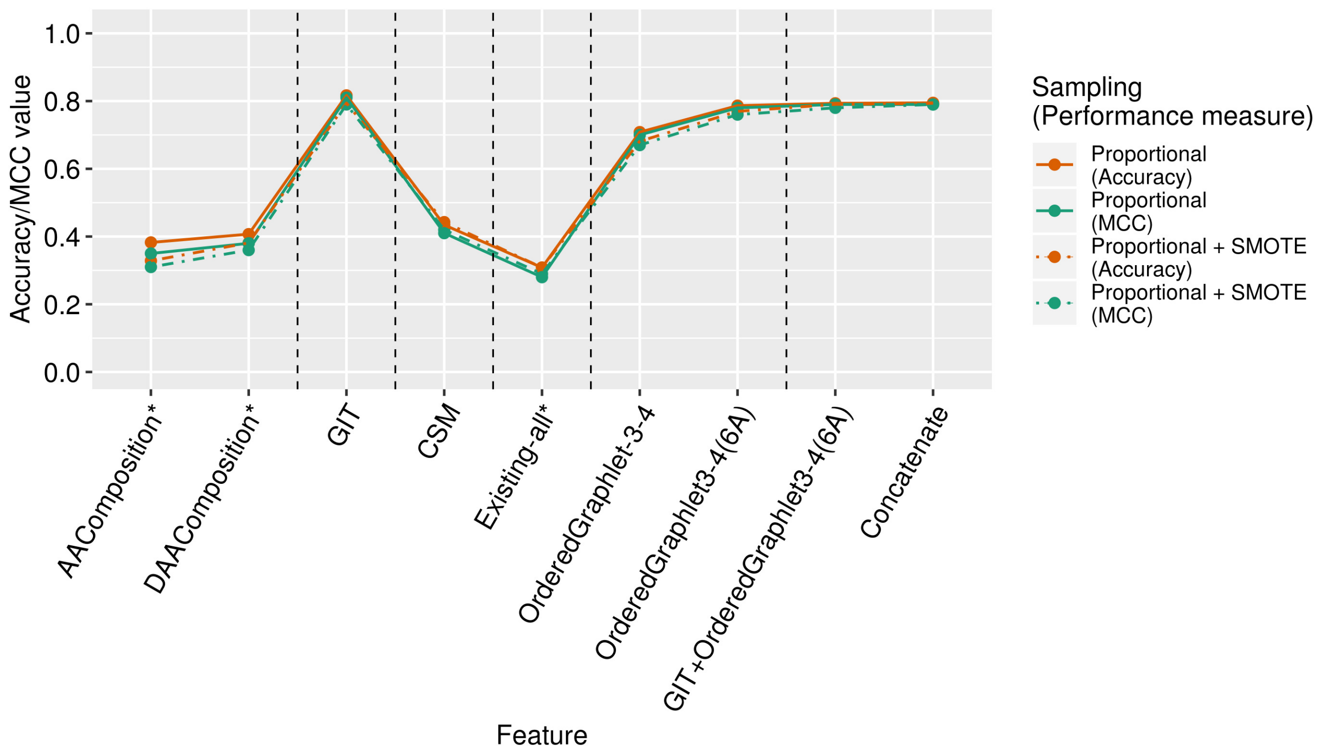

Supplementary Figure S5. Comparison of performance values of features in Figure 3 of the main paper, over all PSN sets in the ASTRAL PSN set. Note that accuracy values are scaled between 0 and 1 to visualize both accuracy and MCC values in the same figure.

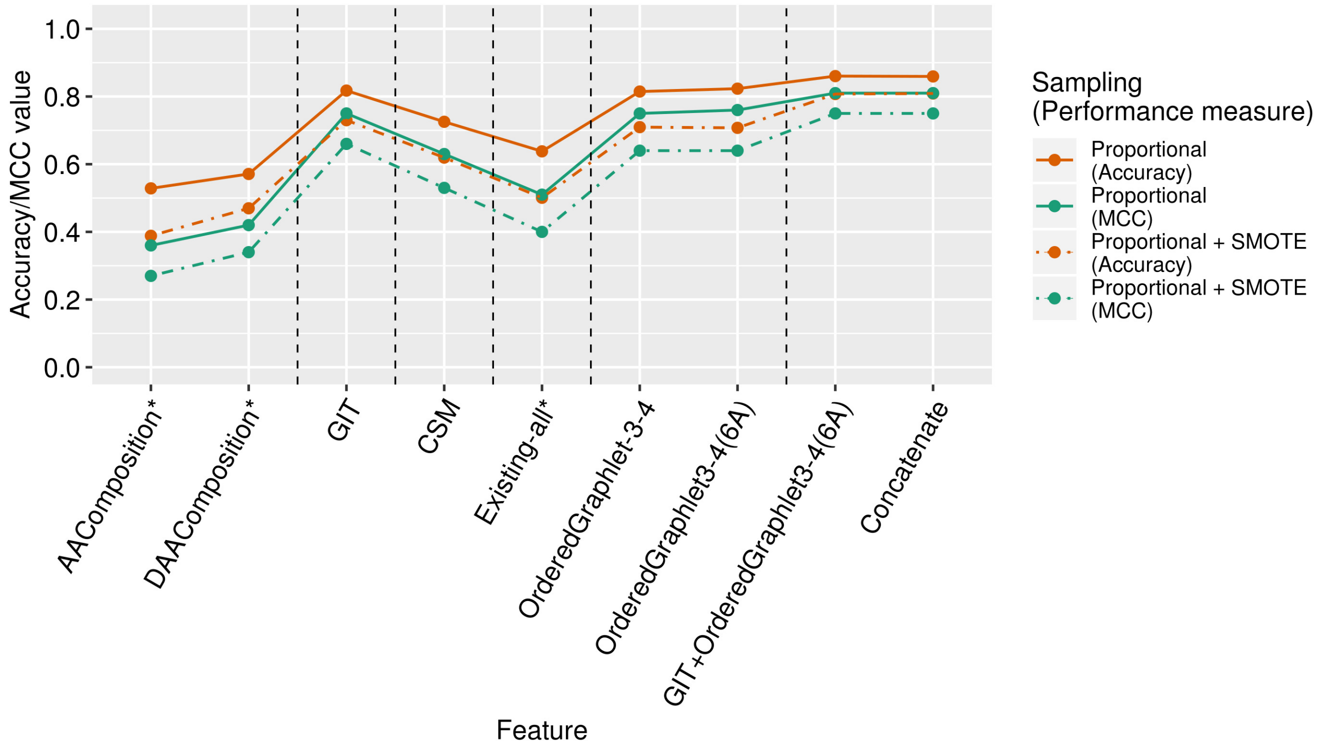

Supplementary Figure S6. Comparison of performance values of features in Figure 3 of the main paper, over all PSN sets in CATH group 1. Results are averaged over all PSN sets in the group (horizontal and vertical lines are standard deviations). Note that accuracy values are scaled between 0 and 1 to visualize both accuracy and MCC values in the same figure.

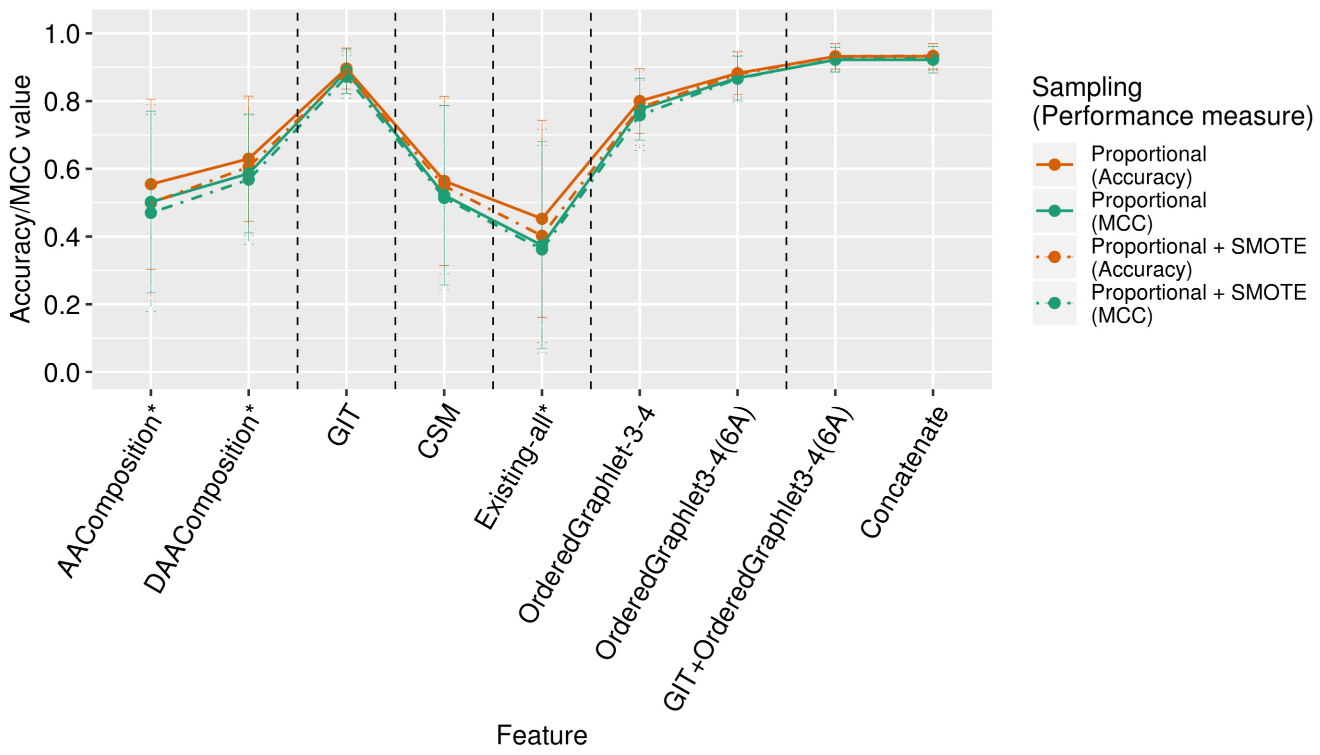

Supplementary Figure S7. Comparison of performance values of features in Figure 3 of the main paper, over all PSN sets in CATH group 2. Results are averaged over all PSN sets in the group (horizontal and vertical lines are standard deviations). Note that accuracy values are scaled between 0 and 1 to visualize both accuracy and MCC values in the same figure.

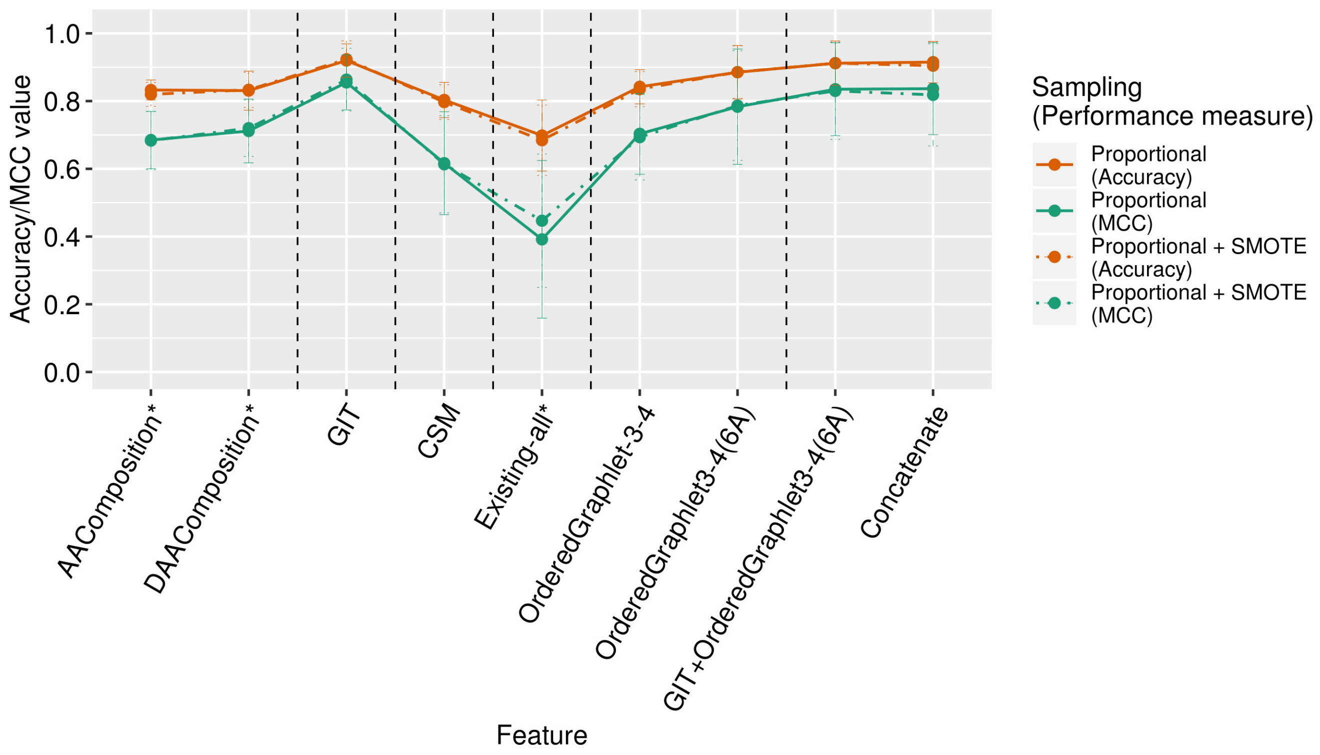

Supplementary Figure S8. Comparison of performance values of features in Figure 3 of the main paper, over all PSN sets in CATH group 3. Results are averaged over all PSN sets in the group (horizontal and vertical lines are standard deviations). Note that accuracy values are scaled between 0 and 1 to visualize both accuracy and MCC values in the same figure.

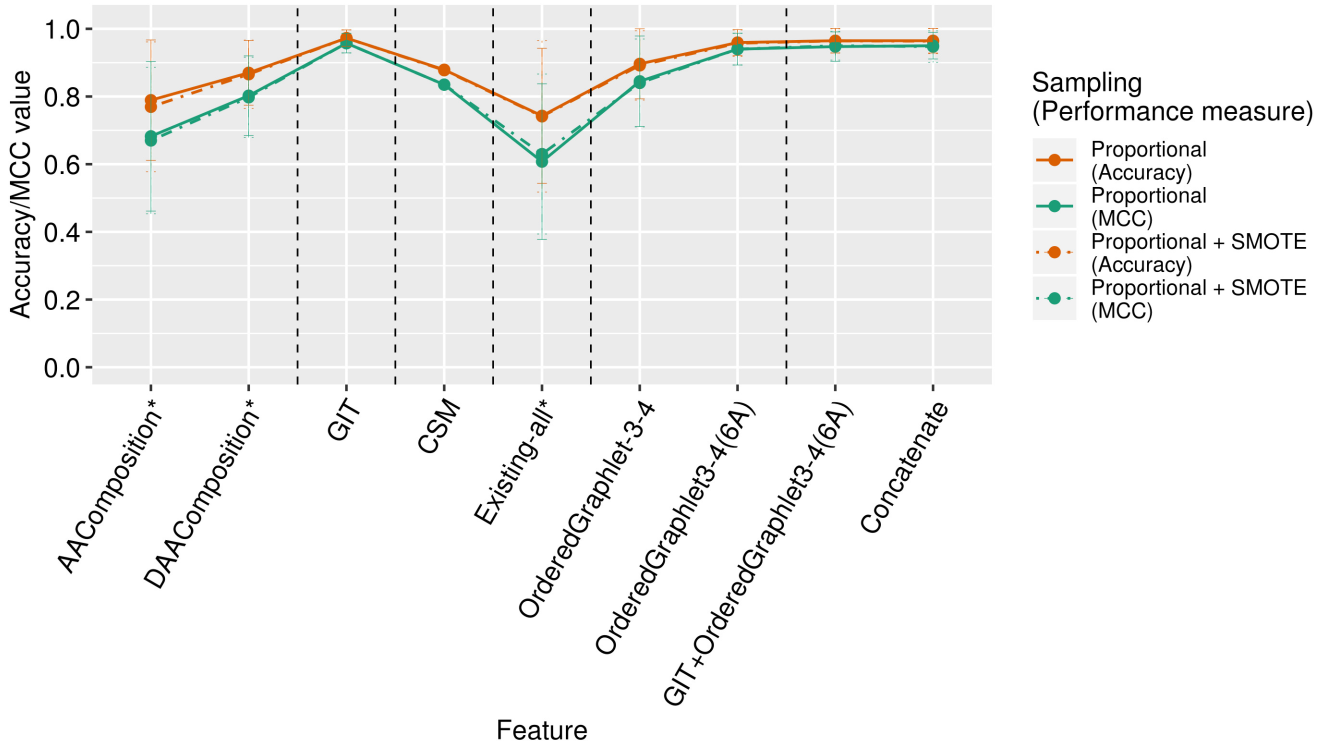

Supplementary Figure S9. Comparison of performance values of features in Figure 3 of the main paper, over all PSN sets in CATH group 4. Results are averaged over all PSN sets in the group (horizontal and vertical lines are standard deviations). Note that accuracy values are scaled between 0 and 1 to visualize both accuracy and MCC values in the same figure.

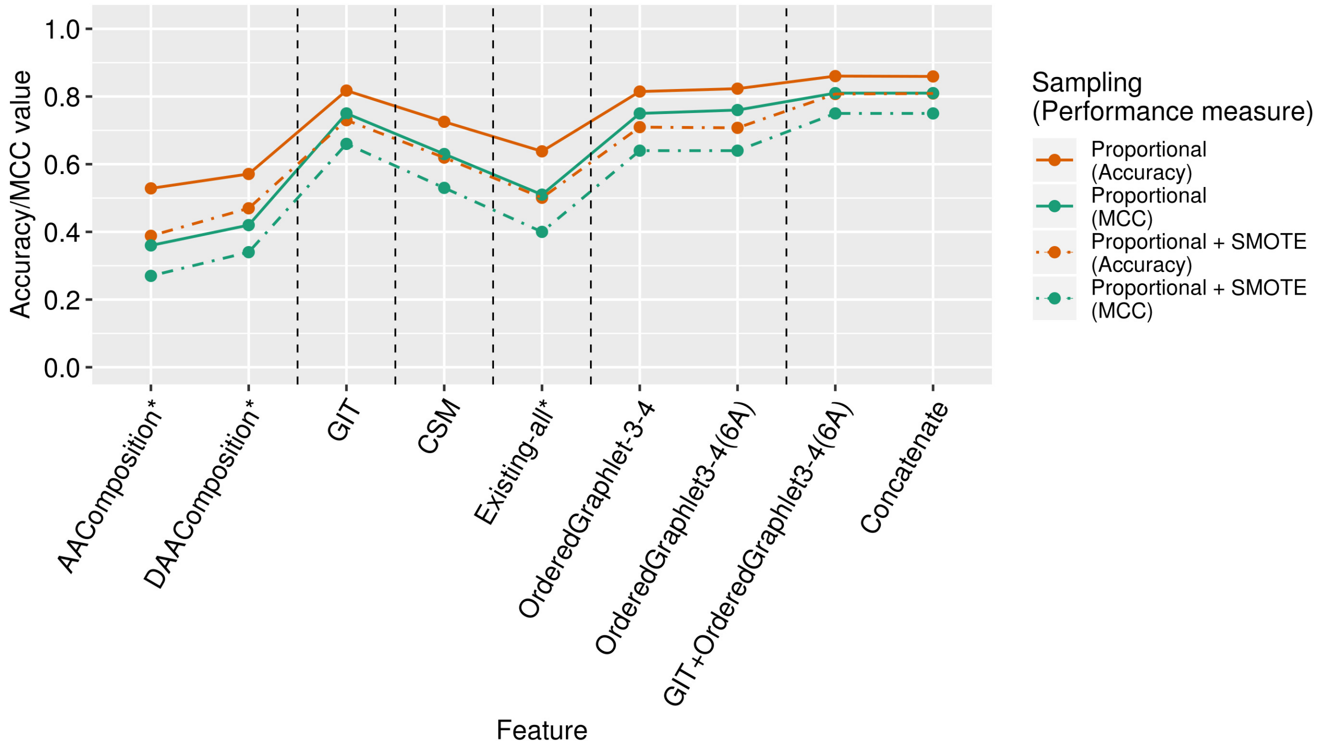

Supplementary Figure S10. Comparison of performance values of features in Figure 3 of the main paper, over all PSN sets in SCOP group 1. Results are averaged over all PSN sets in the group (horizontal and vertical lines are standard deviations). Note that accuracy values are scaled between 0 and 1 to visualize both accuracy and MCC values in the same figure.

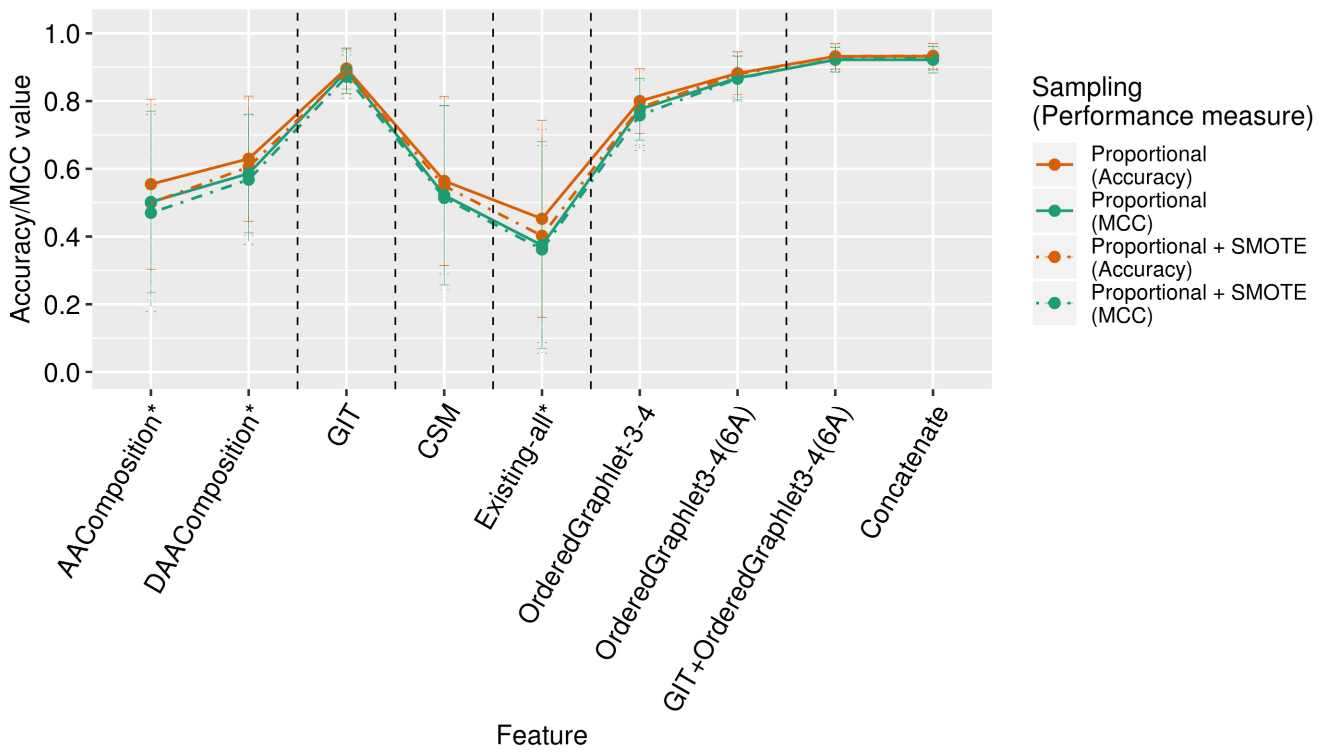

Supplementary Figure S11. Comparison of performance values of features in Figure 3 of the main paper, over all PSN sets in SCOP group 2. Results are averaged over all PSN sets in the group (horizontal and vertical lines are standard deviations). Note that accuracy values are scaled between 0 and 1 to visualize both accuracy and MCC values in the same figure.

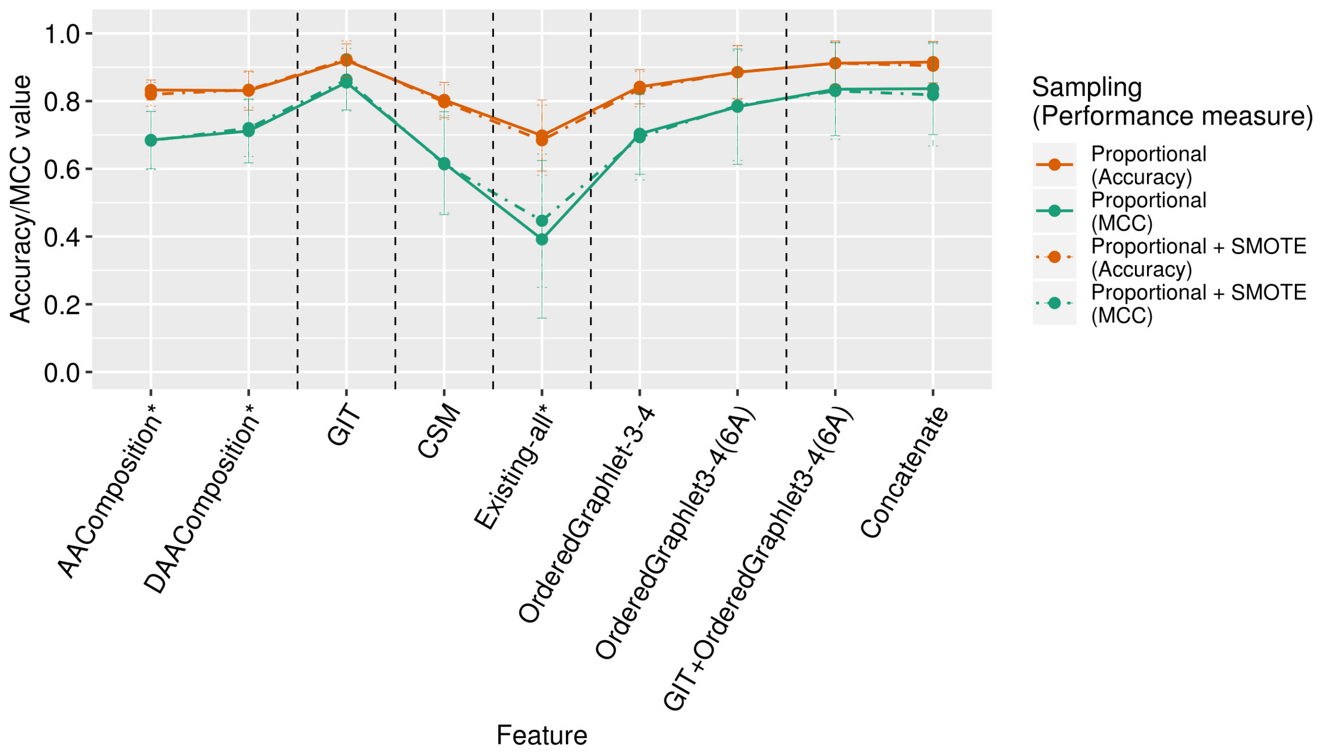

Supplementary Figure S12. Comparison of performance values of features in Figure 3 of the main paper, over all PSN sets in SCOP group 3. Results are averaged over all PSN sets in the group (horizontal and vertical lines are standard deviations). Note that accuracy values are scaled between 0 and 1 to visualize both accuracy and MCC values in the same figure.

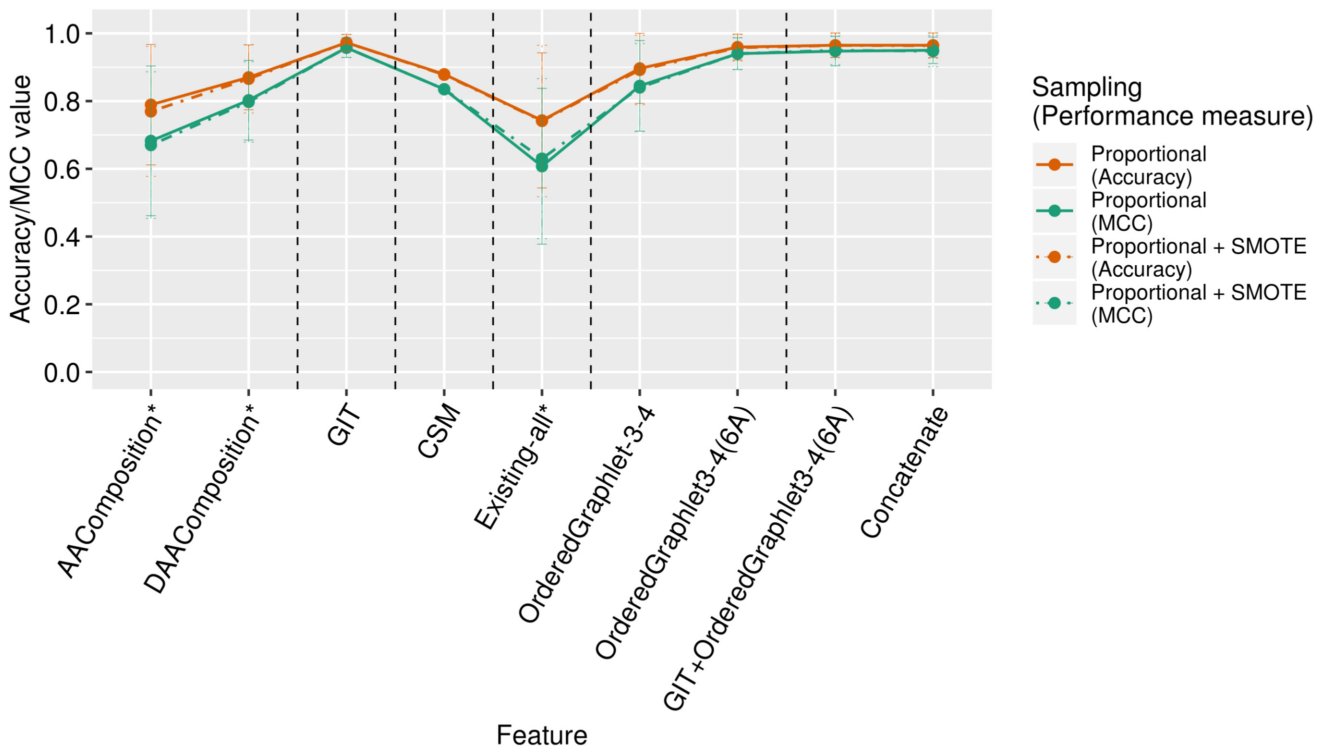

Supplementary Figure S13. Comparison of performance values of features in Figure 3 of the main paper, over all PSN sets in SCOP group 4. Results are averaged over all PSN sets in the group (horizontal and vertical lines are standard deviations). Note that accuracy values are scaled between 0 and 1 to visualize both accuracy and MCC values in the same figure.

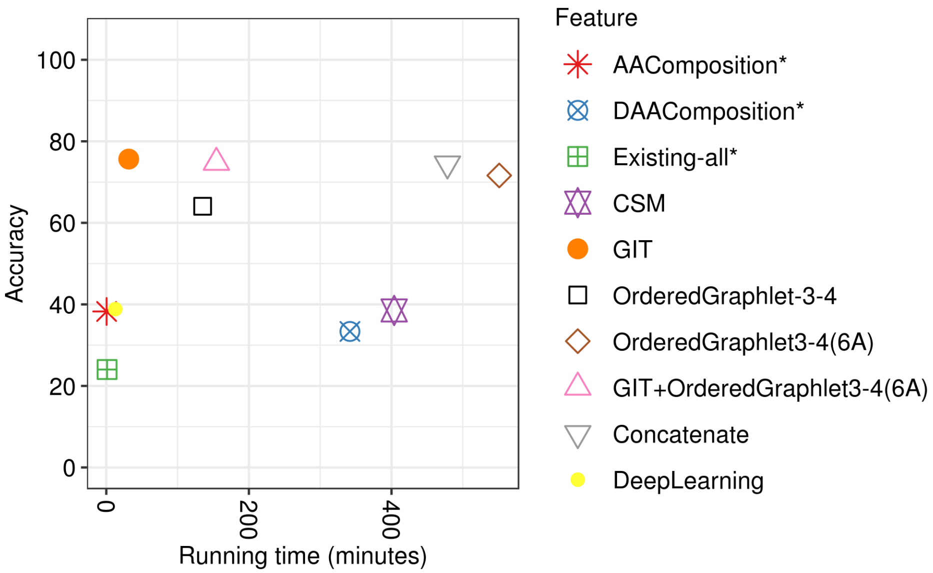

Supplementary Figure S14. Accuracy versus running time of the approaches from Figure 3, for the Astral PSN set, when we do not apply the constraint to exclude the PSNs with a diameter of less than six or the PSNs with fewer than 100 nodes. For each method except DL, the best of its pre- and post-PCA versions is chosen (DL does not have this option). If the latter is selected, "*" is shown next to the given feature's name.

III Supplementary Tables

Supplementary Table S1. Details about our PSN sets belonging to the second-level hierarchical classes of CATH and SCOP. In the table, the first column represents the domain categorization database, the second column represents the PSN set, and the third column represents the different classes present in a PSN set. At the top-level of the CATH hierarchy, there are three classes: α, β, and α/β. At the top-level of the SCOP hierarchy, there are five classes: α, β, α/β, α+β, and Multi domain. Each top-level class has multiple second-level classes, as shown in the table. For example, the α top-level hierarchical class of CATH has four second-level classes: Orthogonal Bundle, Up-down Bundle, Alpha Horseshoe, and Alpha/alpha barrel. For each top-level hierarchical class, we specify its name and the label (separated by semicolon) of that class (the labels correspond to the labels given by CATH/SCOP). For each second-level hierarchical class, we specify its name and the number of PSNs (shown in parentheses) in that class.

	**Top-level hierarchical classes**	**Second-level hierarchical classes**
**CATH**	α, 1	1. Orthogonal Bundle (1632)<br>2. Up-down Bundle (807)<br>3. Alpha Horseshoe (133)<br>4. Alpha/alpha barrel (53)
	β; 2	1. Ribbon (44)<br>2. Roll (242)<br>3. Beta Barrel (699)<br>4. Sandwich (1562)<br>5. Distorted Sandwich (102)<br>6. Trefoil (79)<br>7. 6 Propellor (45)<br>8. 7 Propellor (42)<br>9. 3 Solenoid (70)<br>10. Beta Complex (87)
	α/β; 3	1. Roll (611)<br>2. Alpha-Beta Barrel (839)<br>3. 2-Layer Sandwich (1668)<br>4. 3-Layer(aba) Sandwich (675)
**SCOP**	α; a	1. Globin-like (95)<br>2. Cytochrome c (35)<br>3. DNA/RNA-binding 3-helical bundle (113)<br>4. Spectrin repeat-like (41)<br>5. Four-helical up-and-down bundle (76)<br>6. Ferritin-like (66)<br>7. 4-helical cytokines (38)<br>8. Bromodomain-like (41)<br>9. EF Hand-like (64)<br>10. GST C-terminal domain-like (49)<br>11. SAM domain-like (33)<br>12. Alpha/alpha toroid (53)<br>13. Alpha-alpha superhelix (113)<br>14. Tetracyclin repressor-like, C-terminal domain (35)<br>15. Nuclear receptor ligand-binding domain (30)<br>16. Phospholipase A2, PLA2 (37)
	β; b	1. Immunoglobulin-like beta-sandwich (528)<br>2. Common fold of diphtheria toxin/transcription factors/cytochrome f (85)<br>3. Cupredoxin-like (77)<br>4. C2 domain-like (33)<br>5. Galactose-binding domain-like (68)<br>6. Concanavalin A-like lectins/glucanases (119)<br>7. SH3-like barrel (60)<br>8. PDZ domain-like (39)<br>9. OB-fold (122)<br>10. Beta-Trefoil (61)<br>11. Reductase/isomerase/ elongation factor common domain (39)<br>12. Split barrel-like (33)<br>13. Trypsin-like serine proteases (96)<br>14. Acid proteases (33)<br>15. PH domain-like barrel (83)<br>16. Lipocalins (65)<br>17. 6-bladed beta-propeller (33)<br>18. 7-bladed beta-propeller (35)<br>19. Single-stranded right-handed beta-helix (37)<br>20. Nucleoplasmin-like/VP (viral coat and capsid proteins) (95)<br>21. Double-stranded beta-helix (114)
	α/β; c	1. TIM beta/alpha-barrel (519)<br>2. NAD(P)-binding Rossmann-fold domains (291)<br>3. FAD/NAD(P)-binding domain (102)<br>4. The "swivelling" beta/beta/alpha domain (35)<br>5. Leucine-rich repeat, LRR (right-handed beta-alpha superhelix) (35)<br>6. ClpP/crotonase (38)<br>7. Flavodoxin-like (173)

Supplementary Table S1 – continued on next page

Supplementary Table S1 – continued from previous page

	Top-level hierarchical classes	Second-level hierarchical classes
SCOP	α/β; c	8. Adenine nucleotide alpha hydrolase-like (95)<br>9. Thiamin diphosphate-binding fold (THDP-binding) (45)<br>10. P-loop containing nucleoside triphosphate hydrolases (422)<br>11. Thioredoxin fold (108)<br>12. Anticodon-binding domain-like (31)<br>13. Restriction endonuclease-like (61)<br>14. Ribonuclease H-like motif (211)<br>15. Phosphorylase/hydrolase-like (76)<br>16. PRTase-like (39)<br>17. S-adenosyl-L-methionine-dependent methyltransferases (128)<br>18. PLP-dependent transferase-like (87)<br>19. Nucleotide-diphospho-sugar transferases (42)<br>20. Alpha/beta-Hydrolases (117)<br>21. Ribokinase-like (33)<br>22. Periplasmic binding protein-like I (32)<br>23. Periplasmic binding protein-like II (95)<br>24. Thiolase-like (43)<br>25. HAD-like (61)<br>26. NagB/RpiA/CoA transferase-like (31)
	$\alpha+\beta$; d	1. Lysozyme-like (33)<br>2. Cysteine proteinases (73)<br>3. Ribosomal protein S5 domain 2-like (53)<br>4. Beta-Grasp (ubiquitin-like) (56)<br>5. Cystatin-like (79)<br>6. UBC-like (40)<br>7. Glyoxalase/Bleomycin resistance protein/Dihydroxybiphenyl dioxygenase (45)<br>8. Thioesterase/thiol ester dehydrase-isomerase (56)<br>9. Alpha/beta-Hammerhead (32)<br>10. Ferredoxin-like (213)<br>11. Bacillus chorismate mutase-like (63)<br>12. FwdE/GAPDH domain-like (50)<br>13. Zincin-like (70)<br>14. SH2-like (38)<br>15. Acyl-CoA N-acyltransferases (Nat) (79)<br>16. Profilin-like (55)<br>17. Nudix (31)<br>18. TBP-like (71)<br>19. ATP-grasp (41)<br>20. Protein kinase-like (PK-like) (84)<br>21. Ntn hydrolase-like (63)<br>22. Metallo-hydrolase/oxidoreductase (34)<br>23. Metallo-dependent phosphatases (31)<br>24. LDH C-terminal domain-like (30)<br>25. DNA breaking-rejoining enzymes (34)<br>26. C-type lectin-like (67)<br>27. Nucleotidyltransferase (30)<br>28. Class II aaRS and biotin synthetases (44)
	multidomain; e	1. Beta-lactamase/transpeptidase-like (42)<br>2. DNA/RNA polymerases (84)

Supplementary Table S2. Details about our PSN sets belonging to the third-level hierarchical classes of CATH and SCOP. In the table, the first column represents the domain categorization database, the second column represents the PSN set, and the third column represents the different classes present in a PSN set. At the second-level of the CATH hierarchy, there are nine classes: 1.10, 1.20, 2.160, 2.30, 2.40, 2.60, 3.10, 3.30, and 3.40. At the second-level of the SCOP hierarchy, there are six classes: $a.118$, $b.1$, $c.1$, $c.23$, $c.26$ and $c.55$. Each second-level class has multiple third-level classes, as shown in the table. For example, the 2.60 second-level hierarchical class of CATH has two third-level classes: Jelly-rolls and Immunoglobulin-like. For each second-level hierarchical class, we specify its name and the label (separated by semicolon) of that class (the labels correspond to the labels given by CATH/SCOP). For each third-level hierarchical class, we specify its name and the number of PSNs (shown in parentheses) in that class.

	**Second-level hierarchical classes**	**Third-level hierarchical classes**
**CATH**	Orthogonal Bundle; 1.10	1. Endonuclease III; domain 1 (38)<br>2. Tetracycline Repressor; domain 2 (69)<br>3. Actin-binding protein, T-fimbrin; domain 1 (46)<br>4. Recoverin; domain 1 (58)<br>5. Cytochrome Bc1 Complex; Chain D, domain 2 (47)<br>6. DNA polymerase; domain 1 (65)<br>7. Tetracycline Repressor; domain 2 (69)<br>8. Retenoid X Receptor (51)<br>9. Arc Repressor Mutant, subunit A (97)<br>10. Globin-like (123)<br>11. Cytochrome p450 (42)<br>12. Lysozyme (33)
	Up-down Bundle; 1.20	1. Glutathoine S-transferase Yfyf (Class Pi); chain A, domain 2 (76)<br>2. Butyryl-CoA Dehydrogenase, subunit A; domain 3 (45)<br>3. Fumarase C; chain A, domain 2 (30)<br>4. Methane Monooxygenase Hydroxylase; chain G, domain 1 (56)<br>5. Ferritin (61)<br>6. Four Helix Bundle (120)<br>7. Phospholipase A2 (46)<br>8. Growth hormone; chain A (42)
	3 Solenoid; 2.160	1. UDP N-Acetylglucosamine Acyltransferase; domain 1 (34)<br>2. Pectate Lyase C-like (36)
	Roll; 2.30	1. SH3 type barrels (33)<br>2. Pdz3 Domain (54)<br>3. PH-domain like (70)<br>4. Pnp Oxidase; chain A (46)
	Beta Barrel; 2.40	1. Thrombin, subunit H (123)<br>2. Porin (31)<br>3. Elongation factor Tu; domain 3 (36)<br>4. Lipocalin (102)<br>5.Cyclophilin (32)<br>6. Cathepsin D; subunit A, domain 1 (81)<br>7. OB fold (125)
	Sandwich; 2.60	1. Jelly rolls (507)<br>2. Immunoglobulin-like (932)
	Roll; 3.10	1. Mannose-binding protein A; chain A (75)<br>2. Ubiquitin Conjugating enzyme (39)<br>3. Thiol ester dehydrase; chain A (55)<br>4. Ubiquitin-like (69)<br>5. Endonuclease I-crel (42)<br>6. Nuclear transport factor 2; chain A (85)<br>7. 2-3 Dihydroxybiphenyl 1,2-Dioxygenase; domain 1 (68)
	2-Layer sandwich; 3.30	1. 60s Ribosomal protein L30; chain A (90)<br>2. Ribosomal protein S5; domain 2 (48)<br>3. GMP synthetase; chain A, domain 3 (31)<br>4. Dihydrodipicolinate Reductase; domain 2 (69)<br>5. Enolase-like; domain 1 (93)<br>6. Nucleotidytransferase; domain 5 (177)<br>7. Beta-Lactamase (76)<br>8. Beta polymerase; domain 2 (45)<br>9. D-amino acid aminotransferase; chain A, domain 1 (62)<br>10. SHC adaptor protein (52)<br>11. Alpha-D-glucose-1,6-bisphosphate; chain A, domain 1 (30)<br>12. Heat shock protein 90 (45)<br>13. Alpha-Beta plaits (239)<br>14. Enolase-like; domain 1 (53)
	2-Layer(aba) Sandwich; 3.40	1. Glutaredoxin (154)<br>2. Peroxisomal Thiolase; chain A, domain 1 (71)<br>3. Rossmann fold (412)
**SCOP**	Alph-alpha superhelix; a.118	1. ARM repeat (37)<br>2. TPR-like (32)
	Immunoglobulin-like beta-sandwich; b.1	1. Fibronectin like III (55)<br>2. E-set domains (73)<br>3. Immunoglobulin (304)
	TIM beta/alpha-barrel; c.1	1. (Trans)glycosidases (160)

Supplementary Table S2 – continued on next page

	Second-level hierarchical classes	Third-level hierarchical classes
SCOP		2. Adolase (54)<br>3. Ribulose-phosphate binding barrel (36)<br>4. Metallo-dependent hydrolases (49)
	Flavodoxin-like; c.23	1. CheY-like (41)<br>2. Class-1 glutamine amidotransferase-like (35)<br>3. Flavoproteins (32)
	Adenine nucleotide alpha hydrolase-like; c.26	1. Nucleotidyl transferase (62)<br>2. Adenine nucleotide alpha hydrolase-like (31)
	Ribonuclease H-like motif; c.55	1. Actin-like ATPase domain (88)<br>2. Ribonuclease H-like (92)

Supplementary Table S3. Details about our PSN sets belonging to the fourth-level hierarchical classes of CATH and SCOP. In the table, the first column represents the domain categorization database, the second column represents the PSN set, and the third column represents the different classes present in a PSN set. At the third-level CATH hierarchy, there are six classes: 2.60.120, 2.60.40, 3.20.20, 3.30.390, 3.30.420, and 3.40.50. At the third-level SCOP hierarchy, there are four classes: *b*.1.1, *c*.1.8, *c*.2.1, and *c*.37.1. Each third-level class has multiple fourth-level classes, as shown in the table. For example, the 3.40.50 third-level hierarchical class of CATH has two fourth-level classes: Vaccinia virus protein VP39 and P-loop containing nucleotide triphosphate hydrolase. For each third-level hierarchical class, we specify its name and the label (separated by semicolon) of that class (the labels correspond to the labels given by CATH/SCOP). For each fourth-level hierarchical class, we specify its name and the number of PSNs (shown in parentheses) in that class.

	**Third-level hierarchical classes**	**Fourth-level hierarchical classes**
**CATH**	Jelly rolls; 2.60.120	1. Not yet named (71)<br>2. Jelly rolls (112)<br>3. Not yet named (106)<br>4. Galactose-binding domain-like (82)
	Immunoglobulin-like; 2.60.40	1. C2-domain Calcium/lipid binding domain (36)<br>2. Cupredoxins-blue copper proteins (102)<br>3. Immunoglobulins (501)
	TIM barrel; 3.20.20	1. NADP-dependent oxidoreduxtase (39)<br>2. Aldolase class I (267)<br>3. Glycosidases (184)<br>4. Enolase superfamily (67)<br>5. Metal-dependent hydrolases (58)
	Enolase-like, domain 1; 3.30.390	1. Not yet named (30)<br>2. Enolase-like; N-terminal domain (58)
	Nucleotidyltransferase, domain 5; 3.30.420	1. Not yet named (93)<br>2. Not yet named (53)
	Rossmann fold; 3.40.50	1. Vaccinia virus protein VP39 (175)<br>2. P-loop containing nucleotide triphosphate hydrolase (115)
**SCOP**	Immunoglobulin; b.1.1	1. C1 set domains (antibody variable domain-like) (81)<br>2. V set domains (antibody variable domain-like) (200)
	(Trans)glycosidases; c.1.8	1. Beta-glycanases (53)<br>2. Amylase, catalytic domain (55)
	NAD(P)-binding Rossmann-fol domain; c.2.1	1. LDH-N-terminal domain-like (30)<br>2. Glyceraldehyde-3-phosphate dehydrogenase-like, N-terminal domain (45)<br>3. Alcohol dehydrogenase-like, C-terminal domain (30)<br>4. Tyrosine-dependent oxidoreduxtases (110)
	P-loop containing nucleoside triphosphate hydrolase; c.37.1	1. Nucleotide and nucleoside kinases (48)<br>2. Nitrogenase iron protein-like (30)<br>3. Extended AAA-ATPase domain (40)<br>4. G proteins (111)<br>5. ABC transporter ATPase domain-like (33)<br>6. Tandem AAA-ATPase domain (63)

Supplementary Table S4. Names of the protein domain structural classes where OrderedGraphlet-3-4(6Å) has higher performance than GIT.

Supplementary Table S4

Supplementary Table S5. Confusion matrix for PSN set CATH-2.60.40. We show results only for the "best" (i.e., selected) features.

Features	Labels	2.60.40.10	2.60.40.150	2.60.40.420
GIT	2.60.40.10	483	4	3
	2.60.40.150	2	27	1
	2.60.40.420	4	0	96
OrderedGraphlet-3-4(6Å)	2.60.40.10	482	4	4
	2.60.40.150	6	24	0
	2.60.40.420	2	0	98
GIT+OrderedGraphlet-3-4(6Å)	2.60.40.10	484	4	2
	2.60.40.150	5	25	0
	2.60.40.420	0	0	100
Concatenate	2.60.40.10	485	3	2
	2.60.40.150	5	25	0
	2.60.40.420	1	0	99

Supplementary Table S6. Confusion matrix for PSN set CATH-2.60.120. We show results only for the “best” (i.e., selected) features.

Features	Labels	2.60.120.10	2.60.120.20	2.60.120.200	2.60.120.260
GIT	2.60.120.10	96	0	2	2
	2.60.120.20	1	65	2	2
	2.60.120.200	1	1	97	1
	2.60.120.260	2	4	3	71
OrderedGraphlet-3-4(6Å)	2.60.120.10	96	0	0	4
	2.60.120.20	2	65	1	2
	2.60.120.200	1	0	98	1
	2.60.120.260	4	1	1	74
GIT+OrderedGraphlet-3-4(6Å)	2.60.120.10	98	0	0	2
	2.60.120.20	2	67	1	0
	2.60.120.200	0	0	99	1
	2.60.120.260	2	0	0	78
Concatenate	2.60.120.10	99	0	0	1
	2.60.120.20	2	68	0	0
	2.60.120.200	0	0	99	1
	2.60.120.260	2	0	0	78

Supplementary Table S7. Confusion matrix for PSN set CATH-3.20.20. We show results only for the "best" (i.e., selected) features.

Features	Labels	3.20.20.100	3.20.20.120	3.20.20.140	3.20.20.70	3.20.20.80
GIT	3.20.20.100	29	0	0	0	1
	3.20.20.120	0	56	0	4	0
	3.20.20.140	0	0	48	1	1
	3.20.20.70	2	1	1	222	34
	3.20.20.80	2	1	0	34	143
OrderedGraphlet-3-4(6Å)	3.20.20.100	26	0	0	3	1
	3.20.20.120	0	55	0	2	3
	3.20.20.140	0	0	41	4	5
	3.20.20.70	3	3	6	234	14
	3.20.20.80	1	2	4	16	157
GIT+OrderedGraphlet-3-4(6Å)	3.20.20.100	27	0	0	2	1
	3.20.20.120	0	58	0	1	1
	3.20.20.140	0	0	47	2	1
	3.20.20.70	2	3	0	242	13
	3.20.20.80	0	1	2	11	166
Concatenate	3.20.20.100	27	0	0	2	1
	3.20.20.120	0	58	0	1	1
	3.20.20.140	0	0	47	2	1
	3.20.20.70	2	2	0	244	12
	3.20.20.80	0	1	2	11	166

Supplementary Table S8. Confusion matrix for PSN set CATH-3.30.390. We show results only for the “best” (i.e., selected) features.

Features	Labels	3.30.390.10	3.30.390.30
GIT	3.30.390.10	50	0
	3.30.390.30	0	30
OrderedGraphlet-3-4(6Å)	3.30.390.10	50	0
	3.30.390.30	0	30
GIT+OrderedGraphlet-3-4(6Å)	3.30.390.10	50	0
	3.30.390.30	0	30
Concatenate	3.30.390.10	50	0
	3.30.390.30	0	30

Supplementary Table S9. Confusion matrix for PSN set CATH-3.30.420. We show results only for the “best” (i.e., selected) features.

Features	Labels	3.30.420.10	3.30.420.40
GIT	3.30.420.10	88	2
	3.30.420.40	1	49
OrderedGraphlet-3-4(6Å)	3.30.420.10	88	2
	3.30.420.40	0	50
GIT+OrderedGraphlet-3-4(6Å)	3.30.420.10	88	2
	3.30.420.40	0	50
Concatenate	3.30.420.10	88	2
	3.30.420.40	0	50

Supplementary Table S10. Confusion matrix for PSN set CATH-3.40.50. We show results only for the "best" (i.e., selected) features.

Features	Labels	3.40.50.150	3.40.50.300
GIT	3.40.50.150	169	1
	3.40.50.300	0	110
OrderedGraphlet-3-4(6Å)	3.40.50.150	169	1
	3.40.50.300	0	110
GIT+OrderedGraphlet-3-4(6Å)	3.40.50.150	169	1
	3.40.50.300	0	110
Concatenate	3.40.50.150	169	1
	3.40.50.300	0	110

Supplementary Table S11. Accuracy performance values for each of the considered protein features, for each of the 36 PSN sets, using LR framework.

Supplementary Table S11

Supplementary Table S12. Matthew's Correlation Coefficient (MCC) performance values for each of the considered protein features, for each of the 36 PSN sets, using LR framework.

Supplementary Table S12

Supplementary Table S13. Accuracy and running times (in minutes) of the approaches from Figure 4 in the main paper plus SVMfold, for CATH-3.20.20 and CATH-3.40.50 PSN sets. Due to SVMfold's large time, we could not evaluate it on additional PSN sets.

Approach	Accuracy		Running time (in minutes)	
	CATH-3.20.20	CATH-3.40.50	CATH-3.20.20	CATH-3.40.50
AAComposition*	78.45	79.64	0.06	0.035
DAAComposition*	86.55	86.79	0.51	0.29
GIT	85.86	99.64	2.09	0.35
CSM	77.24	80.71	53.66	15.30
Existing-all*	51.72	60.36	6.42	1.65
OrderedGraphlet-3-4	79.83	97.50	18.70	4.94
OrderedGraphlet-3-4(6Å)	88.45	99.64	167.99	46.95
GIT+OrderedGraphlet-3-4(6Å)	93.10	99.64	170. 08	47.31
Concatenate	93.45	99.64	170.09	47.313
Deep Learning	83.79	92.86	4.99	3.07
SVMfold*	99.31	100	79,365.37	29,859.46

References

1. Faisal, F. E. *et al.* GRAFENE: Graphlet-based alignment-free network approach integrates 3D structural and sequence (residue order) data to improve protein structural comparison. *Scientific Reports* **7**, 14890 (2017).
2. Nelson, D. L., Lehninger, A. L. & Cox, M. M. *Lehninger principles of biochemistry* (Macmillan, 2008).
3. Fan, R.-E., Chang, K.-W., Hsieh, C.-J., Wang, X.-R. & Lin, C.-J. Liblinear: A library for large linear classification. *Journal of Machine Learning Research* **9**, 1871–1874 (2008).
4. Pedregosa, F. *et al.* Scikit-learn: Machine learning in python. *Journal of Machine Learning Research* **12**, 2825–2830 (2011).
5. Chawla, N. V., Bowyer, K. W., Hall, L. O. & Kegelmeyer, W. P. Smote: synthetic minority over-sampling technique. *Journal of Artificial Intelligence Research* **16**, 321–357 (2002).
6. Chicco, D. Ten quick tips for machine learning in computational biology. *BioData Mining* **10**, 35 (2017).
7. Gorodkin, J. Comparing two k-category assignments by a k-category correlation coefficient. *Computational biology and chemistry* **28**, 367–374 (2004).
8. Fadnavis, S. Image interpolation techniques in digital image processing: an overview. *International Journal of Engineering Research and Applications* **4**, 70–73 (2014).
9. Van der Walt, S. *et al.* scikit-image: image processing in python. *PeerJ* **2**, e453 (2014).
10. Glorot, X. & Bengio, Y. Understanding the difficulty of training deep feedforward neural networks. In *Proceedings of the Thirteenth International Conference on Artificial Intelligence and Statistics*, 249–256 (2010).
11. Kingma, D. & Ba, J. Adam: A method for stochastic optimization. *arXiv preprint arXiv:1412.6980* (2014).
12. Abadi, M. *et al.* Tensorflow: Large-scale machine learning on heterogeneous distributed systems. *arXiv preprint arXiv:1603.04467* (2016).
13. Falcon, S. & Gentleman, R. Hypergeometric testing used for gene set enrichment analysis. In *Bioconductor Case Studies*, 207–220 (Springer, 2008).
